# Novel Insights Into Muscarinic and Purinergic Responses in Primary Cultures of Rat Lacrimal Gland Myoepithelial Cells

**DOI:** 10.1167/iovs.62.12.19

**Published:** 2021-09-21

**Authors:** Martin Dankis, Thomas Carlsson, Patrik Aronsson, Gunnar Tobin, Michael Winder

**Affiliations:** 1 Department of Pharmacology, Institute of Neuroscience and Physiology, The Sahlgrenska Academy, University of Gothenburg, Gothenburg, Sweden

**Keywords:** lacrimal gland, myoepithelial cells, muscarinic, purinergic, rat

## Abstract

**Purpose:**

The functional characteristics of receptors that regulate lacrimal gland myoepithelial cells are still somewhat unclear. To date, mainly muscarinic receptors have been of interest; however, further knowledge is needed regarding their expression and functional roles. For this purpose, primary cultures of rat lacrimal gland myoepithelial cells were established and examined functionally.

**Methods:**

Rat lacrimal glands were excised, minced, and further digested, yielding mixtures of cells that were seeded in culturing flasks. After 4-6 weeks, primary monocultures of myoepithelial cells were established, verified by immunocytochemistry. The cells were stained for all muscarinic receptor subtypes (M1–M5) and examined functionally regarding intracellular [Ca^2+^] responses upon activation of muscarinic receptors. For methodological verification, purinergic functional responses were also studied.

**Results:**

Expression of muscarinic receptor subtypes M2-M5 was detected, whereas expression of muscarinic M1 receptors could not be shown. Activation of muscarinic receptors by the non-selective muscarinic agonist methacholine (3 × 10^−11^–10^−3^ M) did not cause a significant increase in intracellular [Ca^2+^]. However, activation of purinergic receptors by the non-selective purinergic agonist ATP (10^−8^–10^−3^ M) caused a concentration-dependent increase in intracellular [Ca^2+^] that could be blocked by the P2 antagonists PPADS and suramin.

**Conclusions:**

Primary cultures of rat lacrimal gland myoepithelial cells were established that displayed a heterogeneous expression of muscarinic receptors. Purinergic functional responses demonstrated a viable cell population. Upon treatment with methacholine, no significant increase in intracellular [Ca^2+^] could be detected, indicating that cholinergic activation of myoepithelial cells occurs via other intracellular messengers or is dependent on interaction with other cell types.

Dry eye disease, also known as xerophthalmia, is a common disorder that globally affects approximately 20% of the population.^1^^,^^2^ Dry eye disease is defined as a multifactorial disease of the ocular surface characterized by a loss of homeostasis of the tear film, accompanied by ocular symptoms in which tear film instability, hyperosmolarity, ocular surface inflammation, surface damage, and neurosensory abnormalities play etiological roles.^3^ The disorder, sometimes associated with anxiety and depression, causes pain and irritation and has been reported to have a significant negative impact on quality of life.^4^^,^^5^

The lacrimal gland consists of three major cell types: the vastly expressed acinar cells from which the majority of lacrimal fluid is secreted; the duct cells, which form the ducts conveying the lacrimal fluid to the surface of the eye; and the myoepithelial cells (MECs), which surround the clusters of acinar cells, together forming so-called acini. The lacrimal gland is densely innervated by parasympathetic nerves,^6^^–^^8^ and loss of parasympathetic innervation has been shown to prohibit normal function of the lacrimal gland.^9^ The major parasympathetic neurotransmitters regulating lacrimal secretion have been shown to be acetylcholine and vasoactive intestinal peptide.^10^ Cholinergic stimuli induce lacrimal secretion from acinar cells via aquaporin-mediated expulsion of water and electrolytes. This occurs in conjunction with exocytotic protein excretion.^11^^,^^12^ MECs, which express α-smooth muscle actin (α-SMA), have been shown to contract upon muscarinic receptor stimulation in salivary glands and mammary glands.^13^^–^^15^ By doing so it has been suggested that they either force expulsion of secrete from the acinar cells or sustain the polarity between the basolateral and apical membrane of the lacrimal gland acini by encapsulating the secretion-induced decreased volume of said stimulated acini. In rat lacrimal myoepithelial cells investigated in non-isolated primary cultures, the non-selective cholinergic agonist carbachol has previously been shown to cause an increase in intracellular [Ca^2+^] with possible observations of subsequent myoepithelial contraction.^16^ This finding was partly supported by studies on intracellular [Ca^2+^] in vivo with two-photon measured responses.^17^ Myoepithelial muscarinic receptors are therefore considered plausible targets for stimulation of lacrimal secretion. A prerequisite of examining this further is verification of muscarinic receptor subtype expression in MECs. Knowledge of subtype expression allows for functional examinations with pharmacological substances with varying selectivity to be conducted.

Purines have recently been suggested to play an important role in lacrimal gland secretion, and purinoceptors have been highlighted as potential targets for pharmacological treatment of dry eye symptoms.^18^ Studies utilizing western blotting and immunofluorescent microscopy have shown that purinergic P2X receptors are expressed in the rat lacrimal gland.^19^ To date, mainly the P2X7 purinoceptor has been suggested as a potential target, as it has shown expression in both rat lacrimal acinar cells and isolated MECs.^20^^–^^23^ Functional responses to P1 purinoceptors have not yet been shown in the rat lacrimal gland, but their expression has been reported in rabbit lacrimal gland acini.^24^^,^^25^

The current study aimed to characterize muscarinic receptor expression and functional responses in primary cultures of MECs. For this, the technique developed by Shatos et al.^26^ and Hodges & Dartt et al.^22^ to isolate and culture primary rat lacrimal gland MECs was utilized. Immunocyto- and immunohistochemistry were used in conjunction to study both primary MEC cultures and whole lacrimal gland tissue slices. For functional characterization of isolated MECs, intracellular [Ca^2+^] was measured upon administration of substances with known selectivity for muscarinic receptor subtypes. For comparison, the non-selective purinergic agonist adenosine triphosphate (ATP) was used to examine functional purinergic responses. To verify the cell cultures, α-SMA, a marker for MECs, was investigated. Further, to verify the absence of pericytes, the expression of cytokeratin 17 (Krt17) was investigated.

## Materials and Methods

### Animals

The study was approved by the Animal Research Ethics Committee in Gothenburg, Sweden (ethical permit #1794/18). A total of 20 adult male Sprague Dawley rats (ages, 10–15 weeks; body weight, 250–400 g) purchased from Charles River Laboratories Italia (Calco, Italy) were used in the study. The study design and experimental procedures followed local rules and regulations at the University of Gothenburg, as well as the ARVO Guidelines for the Use of Animals in Ophthalmic and Vision Research.

### Isolation of Primary MEC Cultures

MECs were isolated from rat lacrimal glands in accordance with a previously described MEC isolation procedure.^22^ Briefly, bilateral lacrimal gland excision was performed in anesthetized (pentobarbitone, 60 mg/kg, intraperitoneally; Kronans Apotek, Sahlgrenska Hospital, Gothenburg, Sweden) rats using disinfected dissection tools. Excised glands were immediately placed in RPMI 1640 medium with glutamine (Lonza, Amboise, France), after which the animals were euthanized by cardiac exsanguination. The RPMI 1640 medium was supplemented with 10 mM HEPES (Thermo Fisher Scientific, Waltham, MA, USA), a cocktail of non-essential amino acids (0.1 mM of each amino acid; Lonza), 1 mM sodium pyruvate (Thermo Fisher Scientific), 2 mM l-glutamine (Lonza), 10 µg/mL penicillin–streptomycin (Thermo Fisher Scientific), and 10% fetal bovine serum (FBS; Sigma-Aldrich, St. Louis, MO, USA). Shortly after, the tissue was minced and subsequently digested in RPMI 1640 supplemented medium containing collagenase type I (0.1%; Sigma-Aldrich) for 1 hour at room temperature. The cells were thereafter further separated through a 70-µm mesh strainer, centrifuged at 300 *g*, plated, and cultured in supplemented RPMI 1640 medium for 4-6 weeks, during which the culture differentiated into a MEC monoculture. During culturing, the cells were continuously investigated through microscopy in conjunction with cell medium replenishment. All subsequent experiments were performed with first-passage cells.

### Immunochemistry

For immunocytochemical investigation, isolated cells (at 4–6 weeks in culture) were trypsinized (5 minutes at 37°C; 0.25% trypsin, 0.02% EDTA, in Hank's balanced salt solution (HBSS; Thermo Fisher Scientific) and transferred to microscopy coverslips, on which they were allowed to settle overnight. The following day the cell medium was discarded, and the cell cultures were fixed in 4% paraformaldehyde (Sigma-Aldrich) in 0.1 M phosphate buffer, pH 7.4 (Sigma-Aldrich) for 10 minutes. The cells were then incubated in phosphate buffered saline (PBS, pH 7.45; Thermo Fisher Scientific) containing 1% normal goat serum (NGS; Vector Laboratories, Burlingame, CA, USA) or normal donkey serum (NDS; Jackson ImmunoResearch Europe Ltd., Cambridgeshire, UK) and 0.1% Triton X-100 (Sigma-Aldrich) for 1 hour at room temperature, for nonspecific background signal reduction. The cells were subsequently incubated for 1 hour with a mouse anti-α-SMA primary antibody (1:250, A5228; Sigma-Aldrich) alone or in combination with either a muscarinic primary antibody (M1–M5) or a primary antibody for Krt17 (1:500, ab109725; Abcam, Cambridge, MA, USA). All primary antibodies were diluted in PBS containing 1% NGS or NDS and 0.1% Triton X-100. The muscarinic primary antibodies were raised in rabbit (M2, 1:100, ab41168, Abcam; M3, 1:100, AB9018, Sigma-Aldrich; M4, 1:100, ab189432, Abcam; M5, 1:100, ab186830, Abcam) or goat (M1, 1:100, ab77098; Abcam). The Krt17 antibody was raised in rabbit. Primary antibody incubation was followed by secondary antibody incubation with Texas Red goat anti-mouse secondary antibody (1:500, ab6787; Abcam), Alexa Fluor 488 goat anti-rabbit (1:250, A32731; Thermo Fisher Scientific), or Alexa Fluor 568 donkey anti-goat (1:250, AB2534104; Thermo Fisher Scientific) for 1 hour at room temperature in PBS containing 1% NGS and 0.25% Triton X-100. A corresponding negative control was run concomitantly for each cell culture by excluding the primary antibody.

Lacrimal glands not used for primary cell culturing were fixed in 4% paraformaldehyde overnight in a refrigerator, after which the tissues were stored in 20% sucrose in PBS until paraffinized and sectioned into 6 µm thick slices (Histocenter AB, Gothenburg, Sweden). In the immunohistochemical experiments, the tissue slices were first deparaffinized, then rehydrated and incubated in sodium citrate buffer (pH 5.0; Sigma-Aldrich) at 95°C for 1 hour. After the heat-induced epitope retrieval, non-specific background block was performed by incubation in PBS containing 0.1% Triton X-100 and 5% NGS for 1 hour. Autofluorescence was reduced by incubation in pH 5-adjusted 5 mM copper sulfate (Sigma-Aldrich) in a 50 mM ammonium acetate (VWR International, Radnor, PA, USA) solution for 10 minutes. The sections were subsequently incubated for 1 hour at room temperature with mouse anti-α-SMA primary antibody (1:250, A5228; Sigma-Aldrich) alone or in combination with a muscarinic primary antibody (M1–M5). All antibodies were diluted in PBS containing 1% NGS or NDS and 0.1% Triton X-100. The muscarinic primary antibodies were raised in rabbit (M2, 1:100, ab41168, Abcam; M3, 1:100, AB9018, Sigma-Aldrich; M4, 1:100, ab189432, Abcam; M5, 1:100, ab186830, Abcam) or goat (M1, 1:100, ab77098; Abcam). The primary antibody incubation was followed by secondary antibody incubation with Texas Red goat anti-mouse secondary antibody (1:500, ab6787; Abcam), Alexa Fluor 488 goat anti-rabbit (1:250, A32731; Thermo Fisher Scientific), or Alexa Fluor 568 donkey anti-goat (1:250, AB2534104; Thermo Fisher Scientific) for 1 hour at room temperature in PBS containing 1% NGS and 0.25% Triton X-100. Each slide contained a corresponding negative control that was exposed to identical procedures with the exception of primary antibody incubation.

All cells and histological slices were mounted with Prolong Gold antifade reagent with DAPI (P36931; Thermo Fisher Scientific) and examined under a Nikon 90i brightfield and fluorescence microscope, and micrographs were recorded utilizing a DS-Fi camera and analyzed with NIS Element 4.40 software (Nikon Corporation, Tokyo, Japan).

### Intracellular [Ca^2+^] Response Measurements

Isolated MEC cultures (at 4–6 weeks in culture) were trypsinized (5 minutes at 37°C; 0.25% trypsin, 0.02% EDTA in HBSS) onto 384-well, optical bottom plates (Thermo Fisher Scientific) and incubated with RPMI 1640-supplemented medium overnight. The following day, the cells were incubated in phenol red-free RPMI 1640 medium supplemented with 10 mM HEPES and 1.2 mM calcium chloride (Sigma-Aldrich) containing FLIPR Calcium 6 (Molecular Devices, San Jose, CA, USA) for 2 hours. Pilot experiments were run in which the cholinergic agonist carbachol (10^−6^–10^−2^ M) was used to examine cholinergic responses. However, since no [Ca^2+^] responses were observed and previous responses to cholinergic stimulation have been attributed to muscarinic receptors, the muscarinic agonist methacholine was chosen instead. During muscarinic stimulation experiments, methacholine (3 × 10^−11^–1 × 10^−3^ M) or corresponding vehicle (HBSS) was administered to MECs. In muscarinic inhibition experiments, the MECs were preincubated with either 4-diphenylacetoxy-*N*-methylpiperidine (4-DAMP, a muscarinic M1/M3/M5-selective antagonist; 10^−9^–10^−7^ M), methoctramine (a muscarinic M2/M4-selective antagonist; 10^−7^–10^−5^ M), *para*-fluorohexahydrosiladiphenidol (pFHHSiD, a muscarinic M3-selective antagonist; 10^−8^–10^−6^ M), or vehicle (HBSS) for 1 hour before administration of the non-selective muscarinic agonist methacholine (3 × 10^−11^–1 × 10^−3^ M) or vehicle (HBSS). During purinergic stimulation experiments, ATP (10^−8^–10^−3^ M) or corresponding vehicle (HBSS) was administered to MECs. In the purinergic inhibition experiments, the MECs were preincubated with the purinergic P2 antagonists suramin (3 × 10^−6^–1 × 10^−3^ M) or pyridoxal phosphate-6-azo(benzene-2,4-disulfonic acid) tetrasodium salt hydrate (PPADS; 10^−6^–10^−2^ M), or with the L-type calcium channel blocker verapamil (10^−8^–10^−5^ M). Intracellular [Ca^2+^] responses were recorded with a SpectraMax i3x fluorescence plate reader (Molecular Devices). During this recording, the isolated MECs were studied functionally by measuring the [Ca^2+^] reagent FLIPR Calcium 6. Increases in intracellular [Ca^2+^] were registered as increases in relative fluorescent unit (RFU), and the corresponding calcium responses were obtained by dividing the maximum RFU by the RFU average of five baseline readings. The baseline readings were measured before administration of any substance or corresponding control (vehicle). All muscarinic and purinergic measurements were concomitantly performed on the same plate with cultured cells from the same animal. All pharmacologically active substances were purchased from Sigma-Aldrich, dissolved in HBSS, and administered utilizing the SpectraMax i3x injector cartridge.

### Statistical Analysis

Statistical significance in the intracellular [Ca^2+^] response measurements was determined by two-way analysis of variance followed by Šídák's test for multiple comparisons. *P* < 0.05 was regarded as statistically significant. Functional data are presented as mean ± SEM. Graphs were generated and statistical analyses were computed using Prism 9 software (GraphPad Software, Inc., San Diego, CA, USA).

## Results

### Isolation of Cell Culture

**Figure 1.**
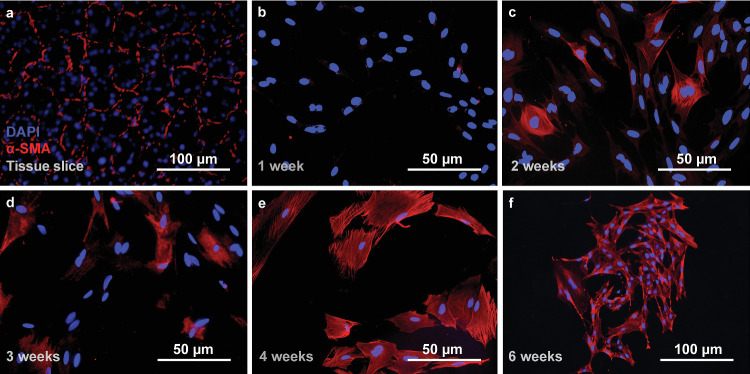
Representative micrographs of α-SMA (*red stain*) in myoepithelial cells. Cells were isolated as primary cultures from rat lacrimal glands. Myoepithelial cells were identified in lacrimal gland tissue slices (**a**), which were then monitored for 6 weeks after cultivation (**b**–**f**). A minute number of MECs were observed in the first week (**b**), followed by a rapid increase during the second week (**c**). In the third week (**d**), there were signs of other glandular cell types dissipating, and by the fourth week (**e**) an isolated myoepithelial primary culture was established. A plateau of MEC proliferation was observed after the fourth week, which was sustained 6 weeks after the initial seeding (**f**). The cell nuclei were visualized with blue DAPI stain. Bars in the lower right corner of the micrographs indicate the scale in each image, respectively.

**Figure 2.**
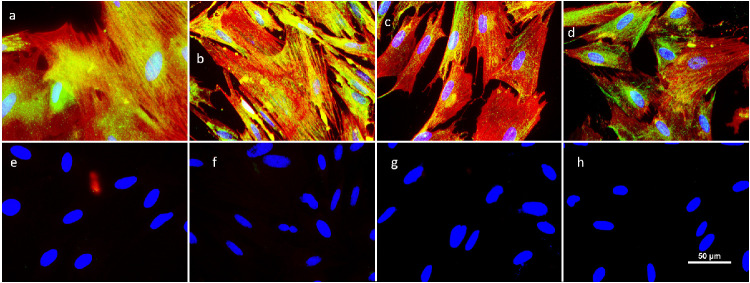
Representative micrographs of Krt17 staining in MEC monocultures. The top panel (**a**–**d**) displays primary cell cultures at 4 weeks after seeding double-stained for α-SMA (*red*) and Krt17 (*green*). Below (**e**–**h**) are the corresponding negative controls. All cells displayed expression of both α-SMA and Krt17, indicating the successful establishment of MEC monocultures. The scale bar applies to all panels.

Primary cultures of MECs were isolated from rat lacrimal glands that displayed an expected histology (Fig. 1a). The cultures reached confluency in the first week (Fig. 1b) and then began to differentiate into MEC monocultures gradually. The MECs, visualized by staining for α-SMA expression, first appeared in minor hubs from which the MECs gradually started to dominate the culture (Figs. 1c, 1d). By weeks 2-3, other cells began to dissipate (Fig. 1d). Isolation of MEC monocultures was confirmed at 4 weeks post seeding (Fig. 1e), which is consistent with previous studies.^22^^,^^26^ The MEC colonization plateaued after week 4, and no noticeable differences were observed between weeks 4 and 6 (Fig. 1f). To further verify the cultures, cells at 4 weeks post seeding were double-stained for α-SMA and Krt17, which enabled differentiating between MECs and pericytes, both of which express α-SMA. The staining showed expression for Krt17 in all cells (Fig. 2), verifying the existence of a monoculture of MECs free from pericytes.

### Functional Studies

**Figure 3.**
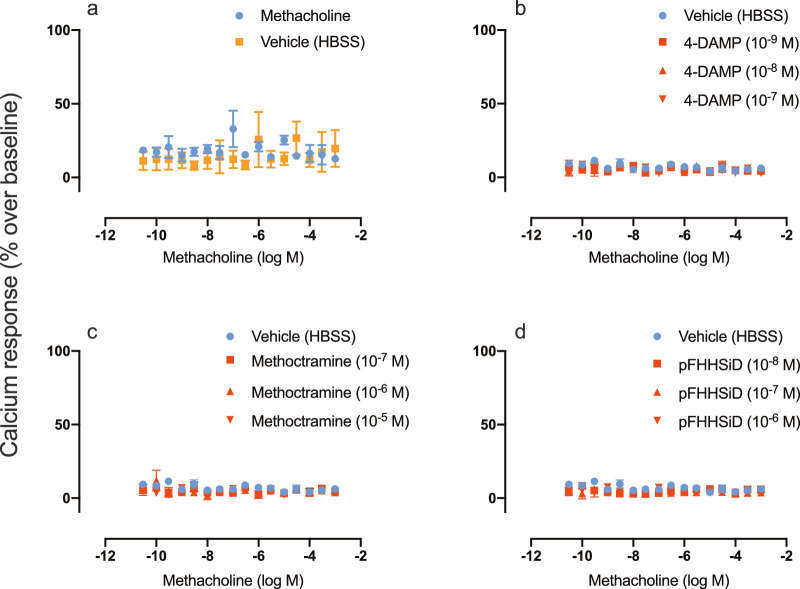
Functional muscarinic receptor responses in isolated primary cultured rat lacrimal gland myoepithelial cells. The *y*-axis represents intracellular [Ca^2+^] responses, shown as percentage over baseline average. The *x*-axis depicts logarithmic molar concentrations of the non-selective muscarinic agonist methacholine. (**a**) Methacholine-induced responses (●, *n* = 5) were measured in conjunction with corresponding vehicle control responses (HBSS, ■, *n* = 5). Methacholine-evoked [Ca^2+^] responses were also measured in cells preincubated with (**b**) the muscarinic M1-/M3-/M5-selective antagonist 4-DAMP (■,10^−9^; ▲, 10^−8^; ▼, 10^−7^ M; *n* = 4), (**c**) the muscarinic M2-/M4-selective antagonist methoctramine (■, 10^−7^; ▲, 10^−6^; ▼, 10^−5^ M; *n* = 4), (**d**) the muscarinic M3-selective antagonist pFHHSiD (■, 10^−8^; ▲, 10^−7^; ▼, 10^−6^ M; *n* = 4), or vehicle (**b**, **c**, **d**; HBSS, ●, *n* = 4). Data points in plots represent mean values of response, and error bars depict SEM.

Morphological and functional characterization was conducted on isolated primary MEC cultures at 4 to 6 weeks post primary seeding. The methacholine-evoked intracellular [Ca^2+^] responses showed no statistically significant differences compared with that of the vehicle solution (average response of methacholine = 18.3% over baseline vs. 14.3% in the HBSS group; *n* = 5) (Fig. 3a). Further, no concentration–response relationship was observed. Functional investigation of methacholine-evoked responses in the presence of muscarinic antagonists 4-DAMP (M1/M3/M5-selective) (Fig. 3b), methoctramine (M2/M4-selective) (Fig. 3c), and pFHHSiD (M3-selective) (Fig. 3d) showed no statistically significant differences in intracellular [Ca^2+^] response as compared with methacholine-induced responses in the absence of antagonists.

**Figure 4.**
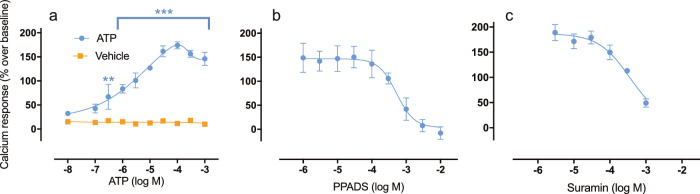
Functional purinergic responses in isolated primary cultured rat lacrimal gland MECs. The *y*-axis represents intracellular [Ca^2+^] responses, shown as percentage over baseline average. The *x*-axis depicts logarithmic molar concentrations of purinergic ligand. (**a**) ATP-induced responses (●, *n* = 4) measured in conjunction with corresponding vehicle control responses (HBSS, ■, *n* = 4). (**b**) Intracellular [Ca^2+^] responses evoked by administration of ATP (10^−5^ M) in cells that were preincubated with increasing concentrations of the purinergic P2X antagonist PPADS (●, *n* = 5). (**c**) Intracellular [Ca^2+^] responses evoked by administration of ATP (10^−5^ M) in cells that were preincubated with increasing concentrations of the purinergic P2 antagonist suramin (●, *n* = 4). Data points in plots represent mean value of response, and error bars depict SEM. ^**^P < 0.01 and ^***^P < 0.001 represent significant differences between vehicle and ATP responses determined by two-way analysis of variance followed by Šídák's test for multiple comparisons.

Purinergic responses were significantly higher than control (*P* < 0.001; *n* = 4) (Fig. 4a). ATP potency was determined as EC_50_ = 1 × 10^−5^ M and efficacy as E_max_ = 170% above baseline. A maximum mean response (174%) was observed following administration of 10^−^^4^ M ATP. ATP stimulation (10^−5^ M) in the presence of increasing concentrations of the purinergic P2 antagonist PPADS resulted in a dose-dependent inhibition of the ATP-induced response (IC_50_ = 5.23 × 10^−4^ M; *n* = 5) (Fig. 4b), and similar dose-dependent inhibition was observed with suramin (IC_50_ = 3.55 × 10^−4^ M; *n* = 4) (Fig. 4c). ATP-induced responses were also measured in the presence of the calcium channel blocker verapamil; however, no effect was observed (data not shown).

### Morphological Studies

**Figure 5.**
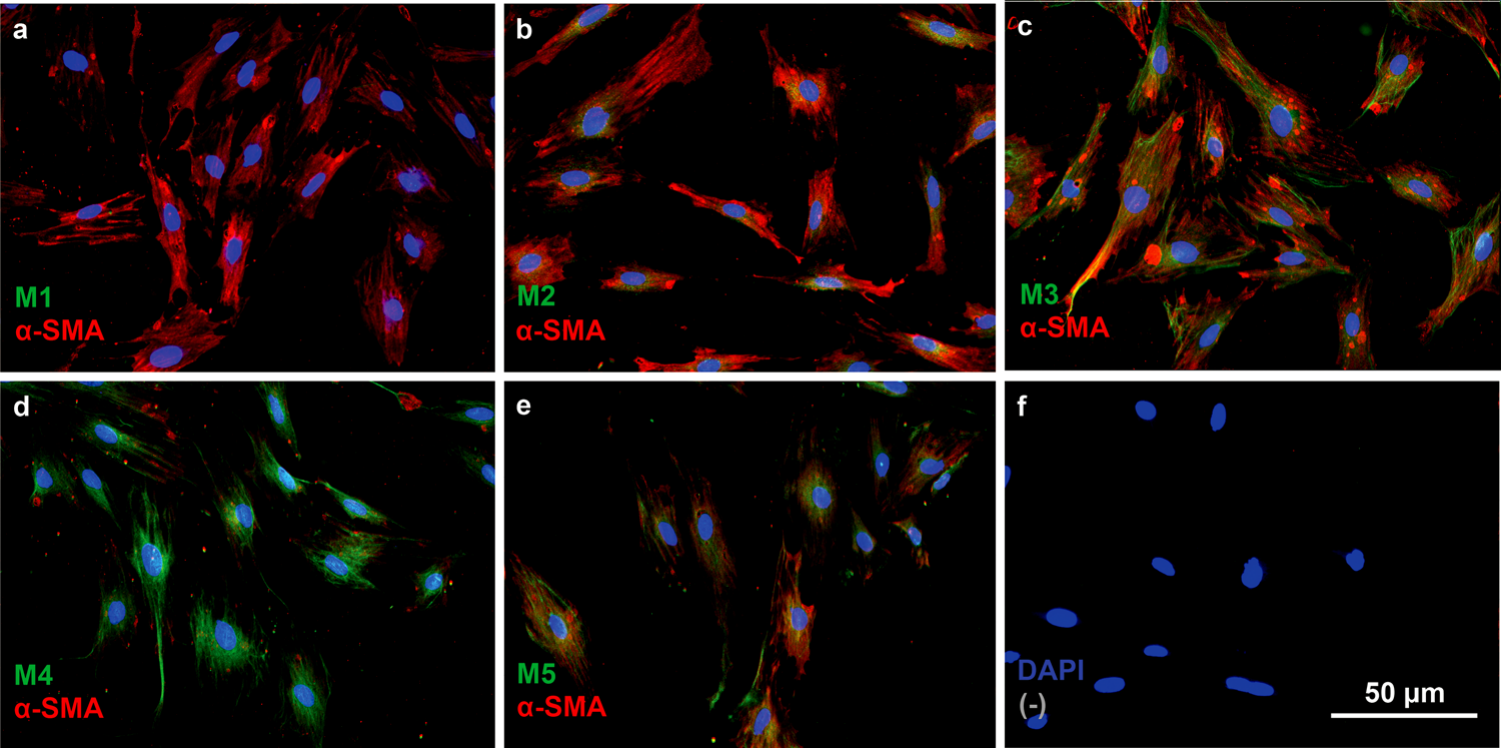
Representative micrographs of muscarinic receptor expression in isolated primary monocultures of rat lacrimal gland myoepithelial cells (*n* = 4). The *red stain* indicates α-SMA expression, and the *green stain* indicates muscarinic receptor subtype expression (M1–M5). The cell nuclei were visualized with blue DAPI stain. (**a**) Absence of fluorescent signal for M1 receptors. (**b**) M2 receptor expression, mainly in close proximity to the nuclei. (**c**) M3 expression in close proximity to the nuclei and microfilament structures. (**d**) Abundant M4 expression. (**e**) Expression of muscarinic M5 receptors. (**f**) Negative control, resulting from exclusion of primary antibody from the immunocytochemistry procedure. The scale bar applies to all panels.

Isolated MECs were investigated for muscarinic receptor subtype expression by fluorescent microscopy. Nuclear staining with DAPI and staining for α-SMA were both visualized concomitantly with staining for muscarinic receptor subtypes M1-M5 (*n* = 4) (Fig. 5). Immunocytochemical staining did not indicate any presence of the muscarinic M1 receptor subtype (Fig. 5a); however, all other muscarinic receptor subtypes (M2–M5) were shown to be present in the MEC monocultures (Figs. 5b–5e). Morphologically, the M2 stain deviated slightly from the others, indicating that the subtype was more densely expressed in the central parts of the cell, seemingly in the membrane above or around the cell nucleus. The stainings for the muscarinic M3, M4, and M5 receptor subtypes displayed more widespread expression throughout the cells. The MEC marker α-SMA was observed in all cells that were investigated for muscarinic receptor subtype expression.

**Figure 6.**
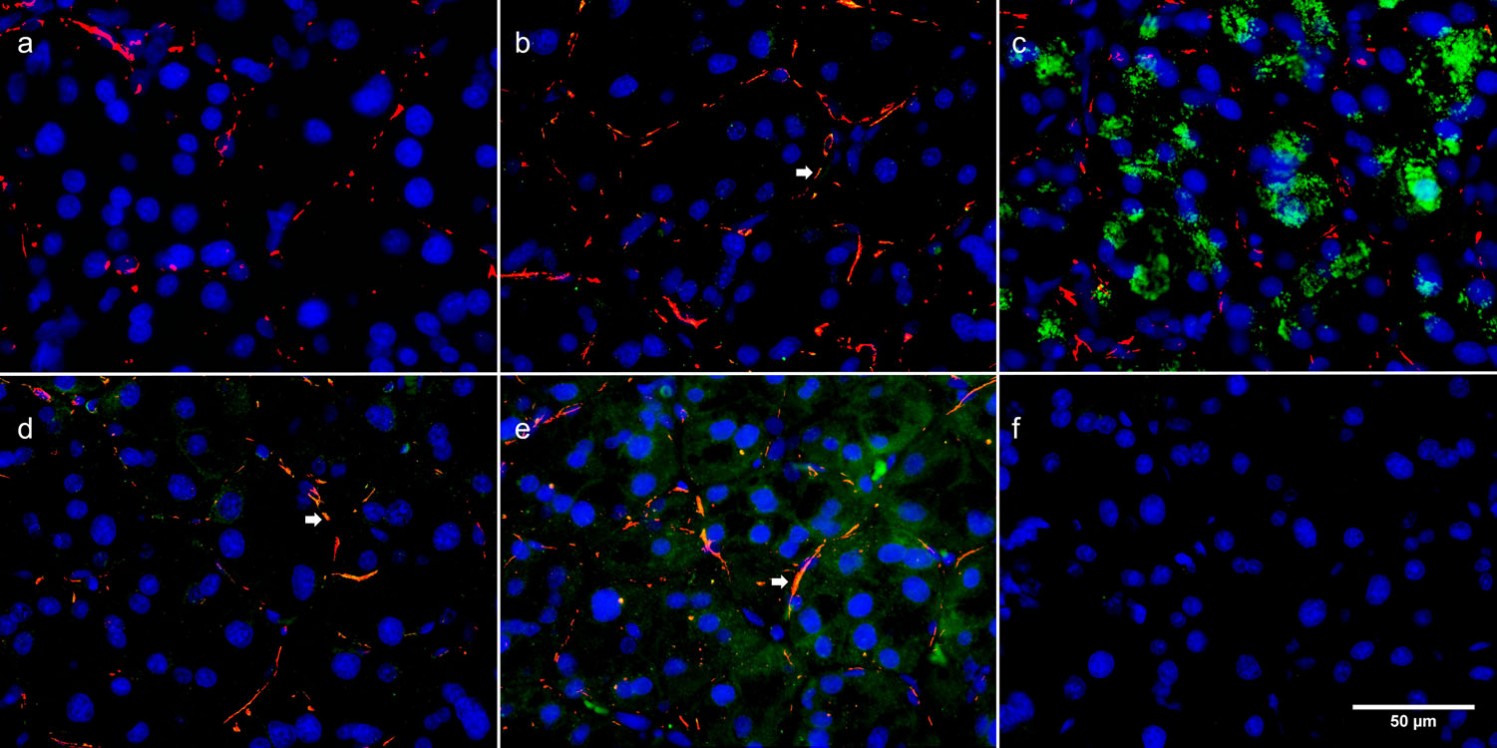
Representative micrographs of muscarinic receptor expression in tissue slices of rat lacrimal gland (*n* = 4). The *red stain* indicates α-SMA expression, and the *green stain* indicates muscarinic receptor subtype expression (M1–M5). The cell nuclei were visualized with blue DAPI stain. (**a**) Absence of fluorescent signal for M1 receptors. (**b**) M2 receptor expression, mainly in myoepithelial cells (indicated by an *arrow*). (**c**) M3 expression, mainly in acinar cells. (**d**) M4 expression, mainly in myoepithelial cells (indicated by an *arrow*). (**e**) Abundant expression of muscarinic M5 receptors, in both acinar and myoepithelial cells (indicated by an *arrow*). (**f**) Corresponding negative control. The scale bar applies to all panels.

Likewise, tissue slices of whole lacrimal gland were investigated for muscarinic receptor subtype expression by fluorescent microscopy. Nuclear staining with DAPI and staining for α-SMA were visualized concomitantly with the expression of muscarinic receptor subtypes M1-M5 (*n* = 4) (Fig. 6). Again, fluorescent staining for the muscarinic M1 receptor subtype was absent (Fig. 6a); however, expression of all other subtypes (M2–M5) was evident. Expression of muscarinic M2 (Fig. 6b) and M4 (Fig. 6d) receptors was seen mainly in MECs, whereas expression of muscarinic M3 receptors was mainly seen in acinar cells (Fig. 6c). Muscarinic M5 expression was abundant in both acinar and myoepithelial structures (Fig. 6e).

## Discussion

In the current study, the successful establishment of primary monocultures of MECs from the rat lacrimal gland was demonstrated. Initially, a co-culture of various cells from the lacrimal gland was established. Then, over time and due to the culturing conditions, a monoculture developed. Interestingly, the current data do not indicate that muscarinic modulation of MEC function is mediated through changes in intracellular [Ca^2+^]. The absence of [Ca^2+^] response following cholinergic stimulation, despite the presence of muscarinic receptors, is somewhat contradictory. The finding could possibly indicate that the cholinergic stimulation is mediated through other second messengers than calcium, such as cAMP or cGMP; however, an increase in intracellular [Ca^2+^] as a response to cholinergic activation has previously been shown in MECs.^16^ It should be noted that these studies were carried out in isolated acini rather than monocultures of MECs. Also, in contrast to the present findings, a recent study in 2- to 3-week-old isolated mouse MEC cultures showed an increase in intracellular [Ca^2+^] following carbachol administration.^20^ The increase in intracellular [Ca^2+^], measured by using Fura-2, was suggested to be followed by a cellular contractile response. The discrepancies between previous data and the current findings may be explained by one of several factors, such as different culturing techniques, different methods to measure intracellular [Ca^2+^], and the fact that previous studies have used the cholinergic agonist carbachol as compared with the muscarinic agonist methacholine used in this study.

We pondered if the absence of net change in intracellular [Ca^2+^] was due to the activities of the excitatory (M1, M3, and M5) and inhibitory (M2 and M4) muscarinic receptors canceling each other out. Utilization of selective muscarinic antagonists allowed for examination of this possibility; however, neither presence of methoctramine (M2/M4), 4-DAMP (M1/M3/M5), nor pFHHSiD (M3) cause any statistically significant change in the [Ca^2+^] responses. This indicates that a nullifying effect is probably not the reason for the lack of an increase in intracellular [Ca^2+^]. Nevertheless, the possibility remains that activation of specific receptor subtypes might have effects on MECs when interacting with acinar cells.

Contrary to the absent methacholine-induced [Ca^2+^] responses, the currently observed increase in intracellular [Ca^2+^] in response to ATP is not surprising. Instead, this is line with previous observations in myoepithelial cells.^18^^,^^20^ However, this is the first time that a clear-cut concentration-dependency has been observed. Further, the induced increase of intracellular [Ca^2+^] could be concentration-dependently blocked with purinergic P2 antagonists. Previous reports have suggested that the purinergic P2X7 subtype is the main propagator of purinergic signaling in the lacrimal gland; however, in the current study a similar conclusion cannot be drawn. Suramin, which dose-dependently blocked the ATP-induced response, is well known for its low affinity to P2X7 receptors as compared with other P2-receptors.^27^^,^^28^ In this regard, it is noteworthy that the ATP-induced [Ca^2+^] response could not be blocked by verapamil. Since verapamil mainly blocks L-type calcium channels, this indicates that the source of [Ca^2+^] is within cellular components, such as the endoplasmic reticulum, or merely that the influx of extracellular [Ca^2+^] is L-type calcium channel independent. Calcium release from the endoplasmic reticulum is typically triggered by inositol 1,4,5-trisphosphate (IP3) formation, which is not what would be expected upon activation of purinergic P2X7-receptors, as these are ligand-gated cation channels. Interestingly, activation of certain P2Y subtypes leads to formation of IP3. However, future studies should be designed to characterize the exact mechanism by which ATP induces [Ca^2+^] responses in MECs.

In the current study, heterogeneous expression of muscarinic receptors in MECs was demonstrated. Expression of muscarinic receptors of the M2, M3, M4, and M5 subtypes was shown through immunocytochemistry, whereas a fluorescent signal for muscarinic M1 receptors was not detected. A similar expression of muscarinic receptor subtypes was seen in whole lacrimal glands. Previously, muscarinic receptor expression has been proposed to be nearly homogeneous in the lacrimal gland.^29^ Studies on acinar cells have strengthened this idea, concluding that they solely express M3 receptors.^30^ Interestingly, it was later shown by the same authors that muscarinic receptors are expressed in both MECs and acinar cells.^31^ The authors consequently reflected upon the need to re-evaluate the assumed cell homogeneity in said preparations, thus implying that homogeneous expression of muscarinic M3 receptors is a characteristic of acinar cells but not necessarily MECs. The current findings strongly support this notion.

Even though the current successful development of primary monocultures has the benefit of allowing studies on isolated MECs, future studies should examine the possibility that cholinergic stimulation is dependent on MEC–acinar interactions. MECs have been shown to be able to act as contractile components in various tissues, such as salivary and mammary glands, thereby aiding in the expulsion of secretory products.13,14,16 Likewise, the MECs in the lacrimal gland have been suggested to contract following calcium depolarization.^26^^,^^32^ However, these observations have been made in acini composites, which contained both MECs and acinar cells. Hence, it is possible that this cholinergically induced depolarization resulted from intercellular transmission, or in acinar cells alone. Interestingly, following administration of a cholinergic agonist, a significant delay in [Ca^2+^] response has been observed in MECs as compared with acinar cells.^16^^,^^33^ This observation raises the possibility that cholinergic activation of MECs is mediated via interaction with acinar cells.

## Conclusions

The current study has demonstrated successful establishment of monocultures of MECs from rat lacrimal glands. Expression of a heterogeneous muscarinic receptor population was shown in the MECs, allowing for the possibility that subtypes other than M3 could be plausible targets for stimulation of secretion from lacrimal glands. Even though activation of muscarinic receptors cannot be shown to cause an increase in intracellular [Ca^2+^], it is possible that the responses are mediated via messengers other than calcium or through intercellular interactions with acinar cells. These intercellular interactions could be dependent on the release of ATP, which clearly induces [Ca^2+^] responses in MECs.
